# Qualitative exploration assessing the acceptability of shared decision-making for prescribing airway clearance techniques in adults with bronchiectasis

**DOI:** 10.1136/bmjopen-2026-119884

**Published:** 2026-07-28

**Authors:** Paul McCallion, Judy Bradley, Joanne Lally, Lisa Robinson, Fiona MacGregor, Anthony De Soyza

**Affiliations:** 1 Newcastle upon Tyne Hospitals NHS Foundation Trust, Newcastle upon Tyne, UK; 2 Population Health Sciences Institute, Newcastle University Faculty of Medical Sciences, Newcastle upon Tyne, UK; 3 School of Medicine, Dentistry and Biomedical Sciences, Queen’s University Belfast, Belfast, Belfast, UK; 4 Population Health Sciences Institute, Newcastle University, Newcastle upon Tyne, UK; 5 Northumbria University, Newcastle upon Tyne, UK; 6 Public Health Sciences Institute, Newcastle University, Newcastle upon Tyne, UK

**Keywords:** RESPIRATORY MEDICINE (see Thoracic Medicine), Chronic airways disease, Decision Making, Physical Therapy Modalities

## Abstract

**Objectives:**

Determine patients’ and physiotherapists’ prospective acceptability of using a shared decision-making (SDM) intervention to support patient choice of airway clearance technique (ACT) prescription for adults with bronchiectasis.

**Design:**

We conducted a qualitative study using focus groups and interviews.

**Setting:**

Participants were recruited throughout the UK. All interviews and focus groups were conducted from the researcher’s private home or Research Team office at Newcastle University. Face-to-face focus groups or interviews were conducted in a university conference room.

**Participants:**

21 physiotherapists and 21 patients living with bronchiectasis participated.

**Results:**

12 themes were identified covering all seven domains of the theoretical framework of acceptability. Themes included: adherence, cultural challenges, education, empowerment, engagement, flexibility, health literacy, overwhelming information, participant understanding, resources, supportive of preferences and time.

**Conclusion:**

A SDM intervention to support patient choice of ACT in bronchiectasis appears to be acceptable to both stakeholder groups. Additionally, we identified novel factors informing the design and implementation of a future SDM intervention. Future research should consider the systematic development of an SDM intervention to support patient choice of ACT in bronchiectasis.

**STRENGTHS AND LIMITATIONS OF THIS STUDY**

- This study provided representation from patients with varying lengths of bronchiectasis diagnosis (6 months to 62 years).
- This study provided representation from respiratory physiotherapists from England, Scotland, Wales and Northern Ireland.
- The use of the theoretical framework of acceptability to evaluate patients’ and physiotherapists’ acceptability of the shared decision-making intervention was appropriate for the aims of the study.
- Most interviews were virtual, therefore ‘missed’ non-verbal cues (eg, participant body language or gestures) influencing the quantity and quality of input from participants, which may have impacted data collection and results.

## Introduction

 Bronchiectasis is a chronic lung condition associated with structural lung damage resulting in a chronic or persistent productive cough typically driven by mucus hypersecretion.^1^ Airway clearance techniques (ACTs) are non-pharmacological approaches to facilitate the removal of mucus in the airways.^2^ ACTs are strongly recommended in international guidelines.^1^

There are a wide range of ACTs used in bronchiectasis including but not limited to manual techniques, a variety of breathing exercises and hand-held devices (ACT adjuncts).^3^ Recent guidelines and meta-analysis conclude there is no single ACT demonstrating superiority over another.^1 4^ Differing aetiology, multimorbidities and fluctuating disease status’ of patients means a single ACT is unlikely to be appropriate for all patients and superior across the disease trajectory.^5^ ACT prescription is complex and prescription patterns differ within and between countries.^3^ ACT prescription can be influenced by mechanisms underpinning different ACTs, availability of ACTs and provider experience.^6^

Shared decision-making (SDM) is an approach where clinicians and patients are encouraged to make treatment decisions together, using the best available evidence.^7^ SDM is particularly relevant where there is no superior treatment recommendation,^8^ such as ACTs in bronchiectasis. SDM interventions are practical materials including patient decision aids (PtDAs) and training packages which are designed to support patients to understand treatment options, benefits and risks of these options and to make informed decisions with their healthcare professional.^9^

Multiple bronchiectasis guidelines recommend personalisation and patient choice when prescribing ACTs.^1 10 11^ The use of SDM is also emphasised in bronchiectasis patient education.^12^ Notably, there are no studies exploring the potential use of an SDM intervention to facilitate patient choice of ACT in bronchiectasis.^13^ However, the development of an SDM intervention to support patients to take an active role in their bronchiectasis care and potentially improve adherence to ACTs has been suggested.^5^

SDM interventions are complex due to their theoretical underpinnings.^14^ Prior to the potential development of an SDM intervention, the Medical Research Council guidance recommends evaluating the anticipated acceptability of key stakeholders (eg, patients and health professionals).^15^

Therefore, we conducted an explorative qualitative study to understand bronchiectasis adult patients’ and respiratory physiotherapists’ anticipated acceptability of an SDM intervention to support patient choice of ACTs in bronchiectasis. Our study used the theoretical framework of acceptability (TFA) to explore key stakeholders (those receiving and delivering the proposed intervention) acceptability of an SDM intervention based on anticipated cognitive and emotional responses.^16^

### Aims

Determine patients’ and physiotherapists’ prospective acceptability of using an SDM intervention to support patient choice of ACT prescription for adults with bronchiectasis.

## Methods

This qualitative study was conducted using a combination of focus groups and semi-structured interviews with patients with bronchiectasis and respiratory physiotherapists.

This study follows the COnsolidated criteria for REporting Qualitative research (COREQ) checklist.^17^

### Patient and public involvement

This study is part of the authors’ larger National Institute for Health and Care Research (NIHR) fellowship on the co-production of an SDM intervention for ACTs in bronchiectasis (reference: NIHR302892). The fellowship had a patient advisory group (PAG). Members of the group (n=3) were recruited from Asthma and Lung UK (ALUK) and UK non-tuberculous *Mycobacterium* (NTM) Patient Networks. These members contributed to the design of the study development and iterations of topic guides (online supplemental material 2). They were not participants in the study. The results of this study were shared with the PAG to facilitate interpretation of findings prior to the next stage in the fellowship.

### Eligibility criteria

#### Patients

Patients were eligible if they were ≥18 years old, diagnosed with bronchiectasis, had capacity to consent and had adequate digital literacy to allow online focus groups/interviews, or had the ability to attend in person focus group/interviews.

Patients were excluded if they had cystic fibrosis related bronchiectasis. Patients were recruited via respiratory charity and patient organisations including ALUK Respiratory Voices, UK NTM Patient Network and the primary ciliary dyskinesia Patient Network. The sampling framework for patients included length of diagnosis, gender, age, employment status, education status and ethnicity.

#### Physiotherapists

Qualified physiotherapists with experience of treating patients with bronchiectasis were eligible. Respiratory physiotherapists throughout the UK were recruited via the Association of Chartered Physiotherapists in Respiratory Care (ACPRC), a UK national body with over 1600 members. The sampling framework for physiotherapists included age, sex, years working in profession and years working with patients with bronchiectasis.

Participants were recruited between December 2023 and March 2024. An advertisement was sent out via newsletters associated with each patient and physiotherapist organisation listed previously. Participants contacted PM via email as an expression of interest. Participant information sheets (online supplemental information file 1) were then sent via email prior to the interviews or focus groups.

### Consent

Fully informed consent was gained by PM prior to commencing interviews or focus groups. Microsoft (MS) digital forms were used for digital consent. Participants were given approximately 1 week between consent and participation in the study.

### Sampling size and sampling

Based on the study aim, theoretical background and quality of dialogue (skills and knowledge of the interviewer, PM), an estimated sample size of 20 per participant group would provide sufficient information power as described by Malterud, *et al*.^18^

Purposeful sampling optimised the study’s limited resources to identify individuals with diverse characteristics, for example, age, gender, geographical location and experience the phenomenon of interest.^19 20^

### Data collection

Physiotherapist and patient focus groups and interviews were conducted separately to ensure each had the space to voice their opinions. Focus groups and interviews were conducted by PM (PhD student and respiratory physiotherapist) and performed virtually or face-to-face. Both physiotherapists and patients were offered a choice. All video calls for interviews and focus groups were conducted from PM’s private home or Research Team office at Newcastle University. Face-to-face focus groups or interviews were conducted in a university conference room.

Topic guides were designed using the TFA questionnaire template by Sekhon, *et al*.^21^ This framework consists of seven domains: affective attitude, burden, ethicality, intervention coherence, opportunity costs, perceived effectiveness and self-efficacy. These reflect to what extent people receiving or delivering a healthcare intervention find acceptable based on anticipated (pre-intervention) or experienced (during or post-intervention) cognitive and emotional responses.^16^ Separate topic guides were designed for respiratory physiotherapists and patients living with bronchiectasis based on reviews of relevant literature and theoretical constructs^13 16 21–23^ (online supplemental information file 2). The concept of SDM was introduced to physiotherapists and patients during interviews. The interviewer read out and shared (either a paper copy or shared screen over a video call) a description of SDM. Topic guides were not tested prior to initial use. Following the first five interviews, transcripts were shared with the PAG and authors’ supervisory team for feedback. Iterations were made to the topic guide for physiotherapists. Interviews and focus groups were audio recorded and transcribed verbatim using a transcription service (https://www.uktranscription.com). Each transcript was assigned a unique study pseudonym.

### Incentives and remuneration

Participants in this study and the project PAG were remunerated for their time and expertise with a £25 voucher or payment as per NIHR payment guidance.^24^

### Data analysis

We used critical realist thematic analysis to analyse the data.^25 26^ This aligned with the overall philosophical position of authors’ wider thesis, critical realism. This approach used qualitative data to explore experiences and views of participants, creating themes and mapping these to an a priori table of constructs outlined by the TFA.^16^ Data were analysed independently by PM and FM (PhD student in forensic radiology). After five transcripts were coded independently, codes underwent standardisation and consolidation by PM and FM. This process occurred again after all transcripts were coded to ensure consistency. Themes were also verified in discussion with the full supervisory team. Themes were mapped to all seven constructs of the TFA. Any discrepancies were mediated through discussion between PM and FM. Coding was managed using NVivo V.14 software.^27^

## Results

42 people (21 respiratory physiotherapists and 21 patients living with bronchiectasis) participated in either focus groups (n=6; 11 physiotherapists, 8 patients) or interviews (n=22; 9 physiotherapists, 13 patients). Participant demographics are displayed in tables 1 and 2. Reason for non-participation for both patients living with bronchiectasis (n=6, 22%) and respiratory physiotherapists (n=7, 25%) was due to not accepting invitation to attend an interview, or a non-attendance at an online interview followed by not replying for an alternative interview date. Time taken for interviews ranged from 28 to 66 min and for focus groups 47–73 min.

**Table 1:** Respiratory physiotherapist participants demographics

Respiratory physiotherapists (n=21)	
Gender (%)	
Male	2 (10)
Female	19 (90)
Ethnic group (%)	
White (English)	10 (47)
White (Scottish)	4 (19)
White (Welsh)	1 (5)
White (Northern Irish)	3 (14)
White (other)	2 (10)
Asian or Asian British (Chinese)	1 (5)
Years working with bronchiectasis patients (%)	
Less than 1 year	1 (5)
1–4 years	2 (10)
5–9 years	4 (19)
10 or more years	14 (66)

**Table 2:** Patient participant demographics

People living with bronchiectasis (n=21)	
Age in years (SD)	66 (12.2)
Gender (%)	
Male	3 (14)
Female	18 (86)
Ethnic group (%)	
White (British)	17 (80)
White (Irish)	1 (5)
White (Roma)	1 (5)
White (other)	2 (10)
Highest level of education (%)	
Secondary school or equivalent	6 (28)
Undergraduate degree	4 (19)
Master’s degree	9 (43)
Doctoral degree	2 (10)
Current employment status (%)	
Employed (full-time)	2 (10)
Employed (part-time)	2 (10)
Unemployed	1 (5)
Retired	16 (75)
Length of diagnosis in years (SD)	9.9 (14.8)
Previously taught airway clearance techniques (%)	
Yes	20 (95)
No	1 (5)

**Figure 1:**
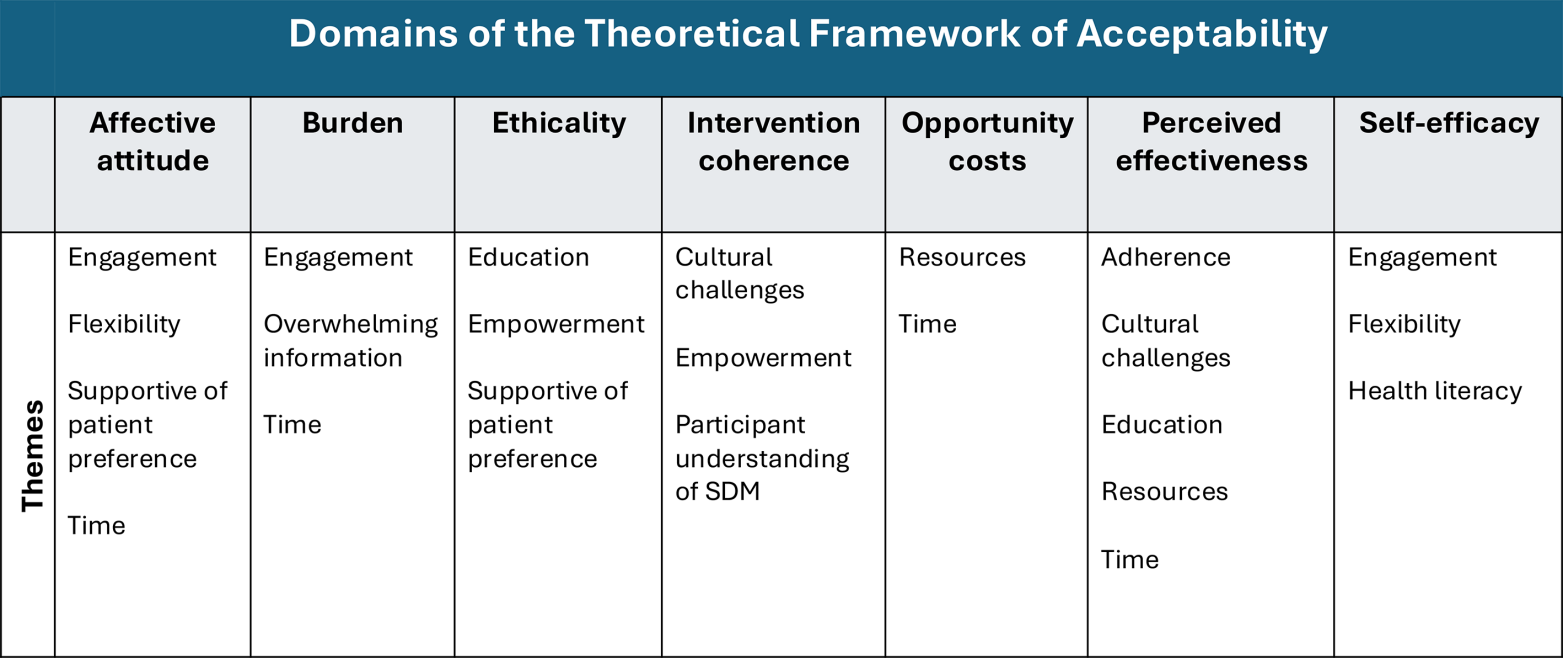
Themes aligned with domains of the theoretical framework of acceptability. SDM, shared decision-making.

12 themes were identified covering all 7 domains of the TFA (figure 1). Each generated theme represents a component of acceptability within the predefined domains of the TFA. Data are described textually in alphabetical order under each thematic heading. Data are presented under thematic headings rather than TFA domain headings to avoid repetition, as some themes related to more than one domain in the TFA. The TFA domain(s) aligned with individual themes are shown beside each subheading. Illustrative participant quotes for every theme are displayed in online supplemental information file 3. Additional quotes for selected themes are displayed in the text. To recognise the difference between participants, patients were given ‘P’ and physiotherapists ‘H’ (ie, healthcare professional). To recognise the difference between interviews and focus groups, ‘I’ or ‘FG’ were added to the end of the identifier. An example for the first interview involving a patient would therefore be ‘PI01’.

### Theme 1: adherence (perceived effectiveness)

Patients’ and physiotherapists felt using an SDM intervention to support choice of ACT may increase adherence to treatment. It was suggested SDM could empower patients to make autonomous decisions on their ACT treatment, and this was specifically associated as a factor to improve adherence by participants (HFG2).

### Theme 2: cultural challenges (perceived effectiveness, intervention coherence)

Patients felt a perceived historical culture of medical-led care may limit the acceptability of some health professionals using SDM, creating a barrier to its implementation:

PI08: It depends. I mean, it depends on who you’re talking to using it [SDM]. I mean, you’re easy to talk to, historically though, really, you’ve got somebody who’s really dominant and saying, “Tch, tch, tch, tch, that’s how you do it,”. If they’re really officious, I wouldn’t like that. It wouldn’t work.

Additionally, there were perceptions that patients were accustomed to a medical-led healthcare culture, and they may fear or resist changing their values or behaviour in consultations, which would be necessary to engage with the SDM intervention (HI02).

### Theme 3: education (ethically, perceived effectiveness)

Patients and physiotherapists discussed how SDM has the potential to educate patients on bronchiectasis in addition to a wider range of ACTs (HI07). Physiotherapists suggested that committing time in initial consultations to educate patients on multiple ACTs using SDM would make future consultations more efficient if there was a need to switch their ACT, as patients would be aware of alternatives (HI05).

### Theme 4: empowerment (ethicality, intervention coherence)

Physiotherapists suggested providing information on multiple ACTs may empower patients to decide on committing to a technique they prefer or what may be best suited to them:

HFG1: I think that the reverse of the overwhelming is the empowering. They’re taking control of, “I’m doing this to treat my bronchiectasis.” So, you’ve given them the information and then they’re saying, “Well, it’s the Acapella that works for me,” or, “The ACBT that works for me.”

Patients highlighted using SDM could support their ability to ask questions within the consultation and feel empowered with their disease management (PFG1).

### Theme 5: engagement (affective attitude, burden, self-efficacy)

Participants felt SDM may improve communication and engagement between patients and physiotherapists. Patients described how they value conversations about what is important to them and their priorities in their bronchiectasis management (PI03). These conversations could then include discussing which ACT may fit best into their routine, and when and where they could perform it.

Participants also highlighted that availability of digital content (website or phone application) may support engagement with SDM and possibly influence their ability to perform their chosen ACT:

PFG1: I don’t mind booklets you know, but could this (SDM) be online to use? To compare clearance exercises [ACTs] I mean…I’ve used BE Happy [a UK patient bronchiectasis group] and the European Lung Clinic websites and things like that…I have found it’s been most informative about the clearance exercises [ACTs].

### Theme 6: flexibility (affective attitude, self-efficacy)

Physiotherapists felt if the choices of ACTs within the SDM PtDA are fixed, this may restrict choice of different ACTs that were not included, which may be better suited for the patient (HI01). Patients discussed there was seldom the flexibility to choose any treatments offered to them in other areas of their health. The ability for ACT prescription to be flexible using SDM was well received by patients (PI02).

### Theme 7: health literacy (self-efficacy)

Participants felt education levels and health literacy to understand the information within an SDM intervention, including the language used by the physiotherapists, would influence acceptability of its use (PFG1). To combat this, in general, participants agreed a key aspect when designing the SDM intervention should be ‘simplicity’ (HFG2).

### Theme 8: overwhelming information (burden)

The potential volume of information that an SDM PtDA may include was an anticipated burden by patients’ and physiotherapists. Both participant groups discussed specific numbers of ACT choices to be included in the PtDA; highlighting that too high a number of ACTs offered may result in patients ‘switching off’ during the consultation, feeling overwhelmed from the information and potentially fail to engage with any ACT:

HFG1: I think one of the cons is overwhelming. We’ve all mentioned that. That they’ll [patients] end up doing nothing because they’ve too much choice.

### Theme 9: participant understanding of SDM (intervention coherence)

Some physiotherapists were unsure what SDM involved. There was an overlap in understanding between ‘shared care’, that is, an agreement of all those involved in someone’s care including the patient, with a particular treatment or plan; and SDM where there is a sharing of information about treatment options, and the patient is supported by the professional to come to a joint decision on which treatment they prefer (HI02). Physiotherapists felt they would need training in SDM (HI03).

### Theme 10: resources (opportunity costs, perceived effectiveness)

Physiotherapists assumed SDM would specifically include an adjunct ACT as an option. Many physiotherapists stated they have no allocated budget for ACT devices (HI05). This prompted discussions of resources as a barrier for using SDM if ACT adjuncts were included.

### Theme 11: supportive of patient preference (affective attitude, ethicality)

Physiotherapists voiced their concerns that using SDM may result in patients selecting the ‘wrong’ ACT if given a choice:

HI06: I guess one of the cons [of SDM] would be that, you know, a patient could choose something that you think might be less effective. But, if they choose that then you, kind of, have to go with it. So, it’s probably just, you know, that catch-22 of giving people options and having to keep your mouth shut even if you think that they’ve chosen the wrong one…

In contrast, other physiotherapists felt using SDM is an ethical intervention for ACT prescription. One physiotherapist felt the prescription of ACTs should be supportive and focus on what the patient is able to do, rather than what the physiotherapist thinks they should do (HFG4).

Patients welcomed the potential opportunity to be educated on alternative ACTs, feel listened to and have their personal preferences considered when choosing their ACT treatment (PI01).

### Theme 12: time (affective attitude, burden, opportunity costs, perceived effectiveness)

Time was a ubiquitous factor of anticipated acceptability of an SDM intervention linking to several domains in the TFA. Some physiotherapists already felt pressured to get through their current bronchiectasis consultation structure, and the thought of adding an SDM intervention into their practice was perceived as a burden or ‘another thing’ to do (HI03). Conversely, other physiotherapists felt SDM would save time in future consultations. They felt using SDM in initial consultations could lead to improved clinical outcomes, empowering patients in their bronchiectasis management and improving patient adherence to their ACT, thereby reducing the need for additional appointments (HFG1).

## Discussion

This is the first study exploring the anticipated acceptability of an SDM intervention to support ACT choice in bronchiectasis. Using a deductive analysis aligned to the TFA domains allowed generation of themes directly associated with factors of acceptability. Themes generated reflected both (1) potential medical and cultural influences on SDM acceptability (time, engagement, cultural challenges) and (2) expectations and challenges when developing the future SDM intervention (table 3).

**Table 3:** Generated themes and their influence for SDM intervention development

Themes	Relevance to SDM intervention development
Education	Patients and physiotherapists felt a patient decision aid should include information to support education on bronchiectasis and a range of ACTs.
Flexibility	Physiotherapists concerned patient decision aid may restrict choice of alternative ACTs. This may influence the number of ACTs to include in the patient decision aid.
Health literacy	Patients and physiotherapists stated they wanted minimal medical jargon in the SDM intervention to support easier readability.
Overwhelming information	Physiotherapists concerned the patient decision aid may provide too much information and overwhelm patients. This may influence the volume of desirable but non-essential content in the patient decision aid.
Resources	Physiotherapists concerned patient decision aid will include multiple ACT adjuncts which they may not be able to provide to patients.

ACT, airway clearance techniques; SDM, shared decision-making.

Most participants in our study felt the use of SDM to support patient choice of ACTs in bronchiectasis was acceptable. One of the main principles of SDM is to facilitate consideration of patient preferences of treatment.^28^ Understanding a patient’s preference and their desire to communicate those preferences is essential for delivering personalised care.^29^ Participants expressed positive views on the proposed use of SDM to provide information on ACTs to elicit patient preference of treatment.

However, the perceived desire or ‘readiness’ of all patients to engage with SDM for ACTs was mixed. To minimise widening healthcare inequalities for those who may not be readily able to engage in SDM, for example, lower educated or older patients, studies recommend flexible formats of PtDAs (eg, multi format or lower readability level PtDAs) should be developed to match individual abilities and preferences.^30 31^

Time is one of the most frequently cited barriers to use of SDM in clinical practice and may reflect one of the most perceived pressures working in the healthcare system.^32 33^ A recent Cochrane review assessed the effects of using PtDAs for people facing health treatment or screening decisions.^34^ The review showed there was no significant difference in consultation time when PtDAs were used in preparation for the consultation (mean difference −2.97 min, 95% CI −7.84 to 1.90; 5 studies, 420 participants). Furthermore, there was a negligible increase in consultation time when PtDAs were used during the consultation (mean difference 1.5 min, 95% CI 0.79 to 2.20; 8 studies, 2702 participants).^34^ Education on the perceived and evidence-based barriers and facilitators to SDM (including time) is an important component of SDM training packages. This may support improved acceptability from physiotherapists using an SDM intervention in clinical practice.

The perceived culture of patients using, and professionals working in the National Health Service (NHS) appeared to be an influencing factor on the acceptability of SDM. This is separate to the lack of readiness or desire by patients described earlier. There are recognised cultural barriers to SDM in healthcare including perspectives on decision role, personal belief systems, trust in clinicians and historical engagement in medical led decision-making.^35^ For example, evidence suggests patients from non-Western cultural backgrounds appraise their personal decision-making process less positively than Western, Caucasian born patients who are the current demographic majority within the NHS.^35 36^ The assumption with SDM is that every patient has an autonomous choice. This is something that may feel uncomfortable or possibly unwanted in non-Western cultures during healthcare consultations.^37 38^

Studies suggest cultural awareness and inclusion of a wider cultural demographic of stakeholders when developing SDM interventions may strengthen its cultural congruency.^35 39 40^ Alden, *et al*^40^ suggest the inclusion of targeted visuals (eg, fonts, colour or images), sociocultural values (eg, the importance of involving family members) and linguistics (eg, the ability to translate material into different languages) may increase the perceived relevance of the treatment decision to be made among different cultures. This may also improve the effectiveness of the PtDA compared with one that has ‘generic’ non-personalised content.^40^ However, Bravo, *et al*^41^ state a balance must be obtained adapting SDM interventions for diverse cultural contexts, without compromising the core essence of SDM.

The potential for SDM to improve patients adherence to ACT was suggested by many participants. Studies have reported an association between participation in SDM and increased adherence to treatment in healthcare,^42–44^ and specifically in respiratory diseases such as chronic obstructive pulmonary disease and asthma.^43 45 46^ However, the need to design and test interventions to support improved adherence in ACTs is highlighted in respiratory guidelines and literature.^10 47 48^ Systematically designing and evaluating the acceptability of any new intervention is essential before testing such hypothesis.^15^

Physiotherapists also highlighted a training need on the use of SDM. A surplus of SDM training packages exist, inclusive of decision coaching and behaviour change techniques.^49^ PtDAs alone have been used to facilitate SDM for healthcare treatments.^50 51^ However, the methods clinicians adopt to take patient histories, compose assessments and articulate findings in a way patients’ can understand and are relevant (where possible) to their preferences and values is essential for SDM.^52^ It is the combination of PtDAs’ and the having the skill to use the information they can elicit (which can be taught with SDM training) that makes an SDM intervention.^49 53^

### Strengths and limitations

There was an attempt to recruit a diverse population of participants. While some diversity elements were successful, for example, a range of educational levels from patients, other elements were not successful, for example, 1/21 physiotherapists were non-white British. This may be reflective of a lack of non-white British physiotherapists working in specialist bronchiectasis clinics, or a lack of non-white British physiotherapists as members of the ACPRC. There was representation from physiotherapists from England, Scotland, Wales and Northern Ireland. Views and experiences were also obtained from patients with varying lengths of diagnosis (6 months to 62 years). Targeted recruitment through specific NHS bronchiectasis services may have allowed a greater opportunity to recruit from a more ethnically diverse physiotherapist and patient population in the UK. Additionally, the study only recruited from the UK, results may have differed and potentially been more widely applicable with a more heterogeneous population.

All patients stated they had a formal diagnosis of bronchiectasis. It is noted that due to recruitment via volunteer and patient networks, we did not have direct access to any personal health records of participants recruited. It has therefore been assumed that patients recruited through the charity and patient organisations above had both clinical and radiological bronchiectasis. There is an increasing prevalence of ‘imposter participants’ in qualitative research, particularly research conducted online.^54^ We aimed to mitigate this risk by recruiting via established patient and professional networks, rather than social media platforms.

### Researcher reflectivity

The researcher was a respiratory physiotherapist by background with an interest in bronchiectasis. There was an aim to balance subjectivity, that is, acknowledgement of the researchers’ interest in the phenomenon of interest (eg, ACTs and bronchiectasis) and objectivity (eg, acknowledging personal assumptions that SDM would be helpful when choosing ACTs). However, this positionality may have monopolised ad hoc questions, prompts or even the tone of voice during interviews which may have influenced participants’ responses and affected the interpretation and analysis of data captured.^55 56^

## Conclusion

The prescription of ACTs is complex. Despite some of the simplified themes generated, there will unlikely ever be a simple method or intervention to prescribe ACTs. The complexity of this problem will rely on some core components within an SDM intervention rather than an exact formula. When designing complex interventions, there will often be unpredictable interactions between contexts (eg, time of day, professional’s or patient’s emotional state) and settings (eg, quiet clinic room or noisy side room of a general practitioner practice) which may determine the success or failure of an intervention.^57^

The development of an SDM intervention for ACTs in bronchiectasis appears to be acceptable to key stakeholder groups and is supported by recommendations laid out in clinical guidelines.^11^ This study provides detailed factors which may influence the future design and implementation of an SDM for ACTs in bronchiectasis. The development of an SDM intervention to support patient choice of ACT in bronchiectasis may result in a user-designed, patient-centred approach to improve knowledge on the role of ACTs, increase adherence to this treatment and improve patients’ long-term quality of life.

## Supplementary material

10.1136/bmjopen-2026-119884 online supplemental file 1

10.1136/bmjopen-2026-119884 online supplemental file 2

10.1136/bmjopen-2026-119884 online supplemental file 3

## Data Availability

Data are available upon reasonable request.
