# Supplementary material for: Qualitative exploration assessing the acceptability of shared decision-making for prescribing airway clearance techniques in adults with bronchiectasis
Source: BMJ Open. 2026 Jul 28;16(7):e119884. doi: 10.1136/bmjopen-2026-119884 (PMC13423159; doi:10.1136/bmjopen-2026-119884)
Supplement: online supplemental file 1 [file bmjopen-16-7-s001.docx]

**
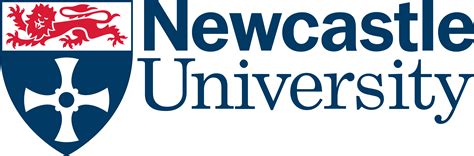

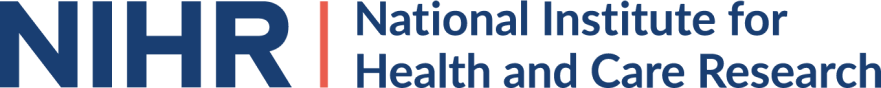
**

**Participant Information Sheet**

**Qualitative exploration of patient and physiotherapists views on Shared Decision Making for Airway Clearance Techniques in adults with Bronchiectasis**

***Introduction***

You are being invited to take part in a research study. Before you decide, it is important for you to understand why the research is being done and what it will involve. Please take time to read the following information carefully and discuss it with others if you wish. If anything is unclear, or if you would like further information, please feel free to ask the person who gave you this leaflet, or speak to a member of the research team directly on 0191 208 8658.

Take time to decide whether you wish to take part.

***What is the purpose of the study?***

This study is aiming to explore the views of patients living with bronchiectasis and of respiratory physiotherapists on shared decision making for airway clearance techniques in adults with bronchiectasis.

Bronchiectasis is a chest condition that can cause repeated infections or “flare ups”. Airway clearance techniques are ways to aid coughing to clear mucus (or sputum) from the lungs. They aim to reduce chest infections and hospital admissions. Guidelines recommend all patients are taught these techniques by a respiratory physiotherapist and then performed at home by the patient (often daily). However, there are many types of airway clearance techniques with little evidence to say which one is best for patients. The best technique may be the one that the patient commits to and uses most regularly.

Shared decision making occurs when professionals and patients are encouraged to make decisions together. This process allows patients to understand treatment options available to them, including the positives and negatives for each option. Shared decision making interventions or “tools” can range from a simple rating scale on a piece of paper to weigh up the pros and cons for each treatment option, to an online interactive tool. It can also help shape the conversations patients have with their physiotherapist to ensure their view and any concerns are heard.


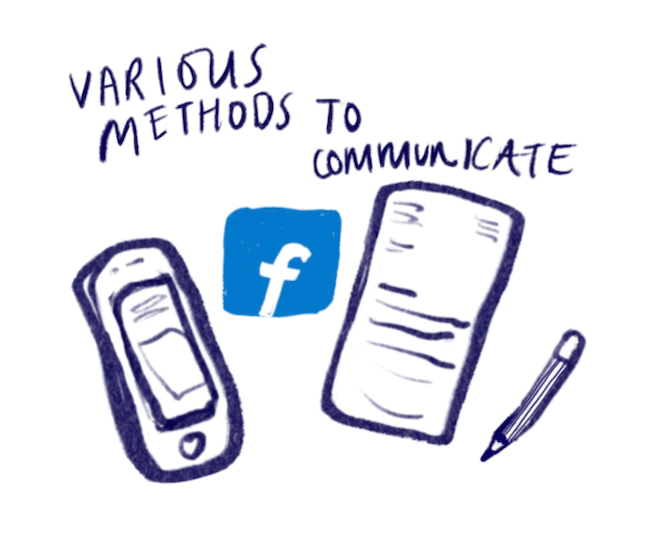


As part of this study, we want to invite you to an interview or focus group with either other people living with bronchiectasis or other respiratory physiotherapists. **People living with bronchiectasis and physiotherapists will be in separate groups to ensure each have the space to voice their views and opinions.** The focus groups will be run by one or two researchers who will ask you and the group some questions about your views on shared decision making for airway clearance techniques in adults with bronchiectasis. We also want to know if you think it would be an acceptable idea to co-develop a shared decision making tool with patients to try and support patient choice of airway clearance techniques for adults with bronchiectasis.

The results of these focus groups and/or interviews will help the researcher and the co-production team design a template for a shared decision making tool; and a training package for respiratory physiotherapists on how to use the shared decision making tool in clinical practice.


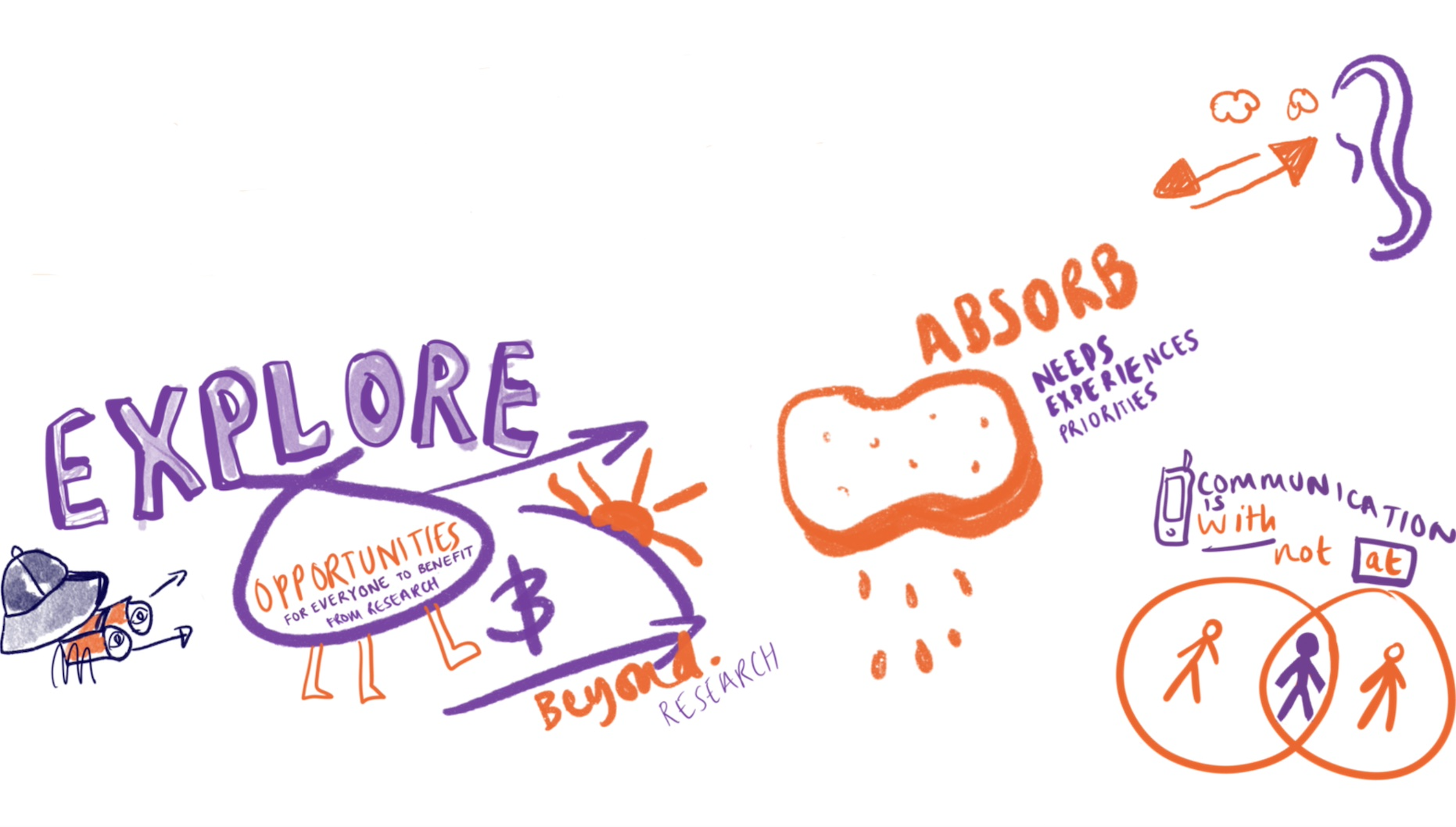


Focus groups will be audio recorded and transcribed so the researchers can spend time analysing your answers. They will last no longer than one hour. This is to make sure your views and opinions have been understood clearly**. You will be offered a £25 voucher for participation in this focus group, or if you prefer an interview**. You are welcome to bring a partner or carer to the focus group or interview if you wish (online or face to face), however they will not be offered a voucher.

***Why have I been chosen?***

You are being asked to participate because you have a diagnosis of bronchiectasis or you are a respiratory physiotherapist who has experience treating people with bronchiectasis.

***Do I have to take part?***

No. It is entirely up to you to decide whether or not to take part. If you do decide to take part you will be asked to sign a consent form, and will be given this information sheet and a copy of the signed consent form to keep (this may be a paper or digital which will be emailed to you). If you decide to take part you are still free to withdraw at any time and without giving a reason. Any data about you that we have collected will remain in the database, unless you ask us to delete it.

***What will happen to me if I take part?***

You will be invited to attend a focus group or interview, which will take place either online (via Zoom or Microsoft Teams) or face to face in Newcastle University. The focus group will contain between 6 to 8 patients with bronchiectasis or respiratory physiotherapists.

If you need support on how to download Zoom or Microsoft Teams to your computer or tablet, please email the lead researcher at **p.mccallion2@newcastle.ac.uk** or telephone call 0191 208 8658.

We will ask a series of questions to you and/or other participants involved in the focus group. The conversations will be audio recorded to be later transcribed for analysis. This recorded information will be stored in a password protected file in the researchers Newcastle University account. Only the research team will have access to the information. Expenses for travel to Newcastle University will be re-imbursed for participants within a reasonable distance of the university. You are welcome to bring a partner or carer to the focus group or interview if you wish (online or face to face).

We will also ask you to give permission to have your details stored in a password protected file held in the researcher’s Newcastle University account to be included on a research ready list to be contacted for future research studies. Your information will be stored for a maximum of three years in line with Newcastle University student guidance. If you agree to be contacted for future studies, we will store your telephone number, address and/or e-mail address on secure file in the researcher’s Newcastle University computer. If an opportunity arises in the future for a clinical study or trial in bronchiectasis that you may be eligible to participate in, the lead researcher would contact you to ask if you wish for further information about the study. You will be under no obligation to take part in this study and any future study would be approved by either Newcastle University or a local research ethics committee.

***What are the possible disadvantages of taking part?***

We do not anticipate any disadvantages to participating in this study.

***What the possible benefits of taking part***

There will be no direct clinical benefit to you from taking part in the study. However, the outcome of the research could influence and meaningfully improve the care of bronchiectasis patients in the future.

***Will my taking part in this study be kept confidential?***

Yes, all information that is collected about you during the course of the research will be kept strictly confidential. The result of the study will not be recorded in your medical notes. No data about individual patients will be published or shared with others.

***What will happen to the data after completion of the study?***

Your data will be kept for 3 years after the study has been completed. After this your data will be permanently deleted.

***What happens if there is a problem?***

If you have any concerns or complaints about your participation in the study you have the right to raise your concern or complaint with the Chief Investigator of the study, Paul McCallion or to the University Supervisor, Anthony De Soyza. You can contact a member of the research team on 0191 208 8658. To ensure participant safety and continual improvements any possible unsatisfactory standards noted during the conduct of the study will be investigated and where required reported to the relevant regulatory authorities.

***What will happen to the results of the research study?***

The results may be presented at patient and charity educational talks, scientific meetings and published in scientific journals. Your name or any other details that may identify you will not be used in any publications.

***Who has reviewed the study?***

Newcastle University Ethics Committee which has responsibility for reviewing research studies at the university has raised no objections to this study.

***Who is organising and funding the research?***

The study is led by Newcastle University. It is funded by the National Institute of Health Research (NIHR) as part of the chief investigators Health Education England (HEE) PhD programme.

***Contact for Further Information***

**Chief Investigator: Paul McCallion**

Newcastle University

p.mccallion2@newcastle.ac.uk

**Supervisor: Professor Anthony De Soyza**

Newcastle University

Anthony.de-soyza@newcastle.ac.uk

Thank you very much for taking the time to read and consider your participation in this study.
