# Supplementary material for: Qualitative exploration assessing the acceptability of shared decision-making for prescribing airway clearance techniques in adults with bronchiectasis
Source: BMJ Open. 2026 Jul 28;16(7):e119884. doi: 10.1136/bmjopen-2026-119884 (PMC13423159; doi:10.1136/bmjopen-2026-119884)
Supplement: online supplemental file 2 [file bmjopen-16-7-s002.docx]

**Introduction (patient focus group / interview)**

- Introduce yourself and confirm consent again verbally with all participants.
- For the purposes of the transcription could everyone introduce themselves, first name only and maybe an interesting fact about yourself or something that you have in the room you are in.
- Explain purpose of the study and this focus group.
  - We want to understand your thoughts and experiences about how your airway clearance technique were taught to you, about the process of shared decision making and how this *could* be implemented into a physio consultation; and finally, your ideas on what a training package for physiotherapists *could* include to support them on how to effectively engage in the SDM process. The interview will take no more than one hour.
- Explain focus group recorded but details will be confidential.
  - I will be recording the session because I don't want to miss any of your comments. People often say very helpful things in these discussions and I can't write fast enough to get them all down. We will be on a first name basis today, and we won't use any names in our reports. You may be assured of complete confidentiality. The recordings will be kept safely in a encrypted file in my university computer account. The audio file only will be sent for transcription. The transcribed notes of the focus group will contain no information that would allow any of you to be linked to specific statements.
- Explain participation
  - This is voluntary.
  - What you tell me will not be discussed with anyone outside of the research team.
  - If there are any questions or discussions that you do not wish to answer or participate in, you do not have to do so
- Explain ground rules
  - Ideally, we want one person to speak at a time. We know there may be a temptation to jump in when someone is talking but please try and wait until they have finished.
  - There are no right or wrong answers – not a test of your knowledge
  - You do not have to speak in any particular order
  - When you do have something to say, please do so.
  - You don’t need to agree with what others say, but please listen respectfully as others share their views
  - Any questions or comments?

**Topic guide – Patients Participants**

1. Can you talk me through how you were offered your airway clearance techniques/ breathing exercises when you were first diagnosed with bronchiectasis?
   1. How much did you feel your airway clearance technique was personalised to you and your specific needs/circumstances?
   2. How many different ways to help clear your chest were you told about or offered when you were diagnosed with bronchiectasis?
   3. Were you asked if the airway clearance technique you were given was acceptable to you?

Before moving on to the next question, I want to discuss what Shared Decision Making is.

Shared decision-making is an approach where clinicians and patients are encouraged to make decisions together, using the best available evidence for treatments. The process is to make sure patients understand the care, support and treatment options available to them, including the positives and negatives associated with each treatment option. These options should be given to patients in a way which is easily understood, without complex medical terminology. The clinician and patient then discuss these options together before coming to a joint decision on which treatment is preferred. So based on that information…

1. How important do you feel being involved in a shared decision making process would be, for deciding your airway clearance technique?
   1. How confident do you feel you could engage in a shared decision making process with your physiotherapist?
   2. How might patients struggle to engage in shared decision making with their physiotherapist? How could these barriers be navigated or supported?
   3. What might be some/any negative impacts for patients engaging in shared decision making for their airway clearance?
      1. Do you feel this/these would outweigh the potential benefits of using shared decision making?
   4. How might your adherence with your airway clearance on a regular basis change, if you were involved in a shared decision making process with your physiotherapist when choosing which technique you would prefer to use?
   5. Would there be any circumstances where you feel shared decision making would not be appropriate within a physiotherapy consultation/appointment? If answers: Can you tell me more about that? Why?
   6. Has anyone /you been involved in shared decision-making discussions for any other part of their / your care for their bronchiectasis? If yes, Can you tell me more about that?
2. What information on airway clearance techniques/ breathing exercises were you provided before you attended your first physiotherapy appointment?
   1. How did you use that information? (e.g. did it help in your consultation with the physiotherapist? If so, how? If not why?)
   2. Did you/anyone search for any information yourself before your physiotherapy appointment? Google/search engine? Can you tell me more about that or why you decided to do that?
3. Looking back, what information do you think would have been useful for your physiotherapist to share with you - about the airway clearance techniques (available) to help you decide which one you might prefer to use?
   1. What might be the best way to show or present (this information?) information on airway clearance techniques with patients?
   2. If this information was presented to you, what type of format do you think this should be? Paper, digital, both, other?
   3. Would a video or drawings showing each/a range of airway clearance techniques/ breathing exercises be useful? Would it help support your choice more than words alone – how/why?
   4. How many airway clearance options/choices of techniques do you think would be acceptable to offer during the appointment?
   5. Again looking back, if you could be given information on airway clearance techniques, would you want this before attending your physiotherapy appointment, during or after your appointment?
4. Can I ask you now to think about overall, when considering all the things you've spoken about, what did you think about your initial bronchiectasis physiotherapy experience?
5. Overall, do you think the use of SDM to support patients choose their ACTs would be acceptable? (Y/N).
6. What else do you feel would help support your airway clearance management for your bronchiectasis?

**Introduction (Physiotherapy interview/focus groups)**

- Introduce yourself and confirm consent again verbally.
- Explain purpose of the study and this interview.
  - We want to understand your thoughts and experiences about how ACTs are prescribed, about the process of shared decision making and how this *could* be implemented into a physio consultation; and finally, your ideas on what a training package for physiotherapists would include to support them on how to effectively engage in the SDM process. The interview will take no more than one hour.
- Explain interview recorded but details will be confidential.
  - I will be recording the session because I don't want to miss any of your comments. People often say very helpful things in these discussions and we can't write fast enough to get them all down. We will be on a first name basis today, and we won't use any names in our reports. You may be assured of complete confidentiality. The recordings will be kept safely in a encrypted file in my university computer account. The audio file only will be sent for transcription. The transcribed notes of the interview will contain no information that would allow you to be linked to specific statements.
- Explain participation
  - This is voluntary.
  - What you tell me will not be discussed with anyone outside of the research team.
  - There are no right or wrong answers – not a test of your knowledge
  - If there are any questions or discussions that you do not wish to answer or participate in, you do not have to do so
  - Any questions or comments?

**Topic guide – Physiotherapists Participants**

1. Can you talk me through the components of a bronchiectasis physiotherapy consultation for a patient?
2. What is the main driver for whichever airway clearance techniques you prescribe to patients?
   1. BTS/National Guidelines, patient/clinician preference?, BSI, evidence base, clinical/department budget
   2. Why is that the main driver for you?
3. What may be barriers to offering multiple/a range of airway clearance techniques to patients in your clinic/ward?
   1. Prompts - time, resources, physiotherapy skill set, access to adjuncts, other?
   2. How do you think these barriers could be overcome?
4. Can you discuss what you feel shared-decision making is and what it involves?

**Give definition below to support their answers to the proceeding questions.**

Before moving on to the next question, I want to discuss what Shared Decision Making is. I know there was some brief information to you on this within the study information sheet. Shared decision-making is an approach where clinicians and patients are encouraged to make decisions together, using the best available evidence for treatments. The process is to make sure patients understand the care, support and treatment options available to them, including the positives and negatives associated with each treatment option. These options should be given to patients in a way which is easily understood, without complex medical terminology. The clinician and patient then discuss these options together before coming to a joint decision on which treatment is preferred. So based on that information…

1. How might shared decision-making support patients choose which airway clearance technique may be best for them?
   1. How do you think using shared decision making to support patients’ choice of airway clearance technique might influence their adherence?
2. What do you feel would be the pros and cons of a shared decision-making approach if this was adopted into bronchiectasis clinical practice?
   1. Lengthy appointments, opportunity cost to other aspects? PR/meds, lack of pt engagement, teaching physios how to use it?
   2. Improved pt satisfaction, empowerment, better education? PROMS/?clinical improvements/adherence.
3. What do you feel are the key pieces of information to share with patients around their airway clearance techniques?
   1. how would you differentiate between two techniques, how would you explain that to a patient?
   2. What type of format do you think information should be shared with for patients? Paper, digital? Both other? Multi-lingual? Pictures? Videos?
   3. If sharing information on a range of airway clearance techniques, how many choices/ACTs do you think would be acceptable?
   4. Prompt if not covered: Would a video showing each/a range of airway clearance techniques/ breathing exercises be useful?
4. Have you had any shared decision-making training to date?
5. Thinking about how you and other bronchiectasis physiotherapists might learn about shared-decision making and how to use it in clinical practice to support patients’ choice of airway clearance, what method(s) of training do you feel would work for this?
   1. F2F workshops? Online tutorial? Webinar? Peer to peer? One “champion” at the trusts and dissemination through in-service/ micro teaching?
   2. What do you feel would be the key things to include in this teaching/training package for shared decision making on airway clearance techniques?
6. Overall, do you think the use of SDM to support patients choose their ACTs would be acceptable? (Y/N).
7. What else do you feel would help support patients to optimise their concordance with airway clearance for their bronchiectasis?

**Topic guide – Physiotherapists Participants (Version 2 – iteration)**

1. Can you talk me through the components of a bronchiectasis physiotherapy consultation for a new patient?
   1. ***“New” was added rather than review to allow a more detailed response for everything that would be included in a physio consultation. The PAG felt the SDM may be primarily used primarily for NPs and so it important to understand the details of a new assessment.***
2. What is the main driver for whichever airway clearance techniques you prescribe to patients?
   1. BTS/National Guidelines, patient/clinician preference, BSI, evidence base, clinical/department budget
   2. Why is that the main driver for you?
3. What may be barriers to offering multiple/a range of airway clearance techniques to patients in your clinic/ward?
   1. Prompts - time, resources, physiotherapy skill set, access to adjuncts, other?
   2. How do you think these barriers could be overcome?
4. Can you discuss what you feel shared-decision making is and what it involves?

**Give definition below to support their answers to the proceeding questions.**

Before moving on to the next question, I want to discuss what Shared Decision Making is. I know there was some brief information to you on this within the study information sheet. Shared decision-making is an approach where clinicians and patients are encouraged to make decisions together, using the best available evidence for treatments. The process is to make sure patients understand the care, support and treatment options available to them, including the positives and negatives associated with each treatment option. These options should be given to patients in a way which is easily understood, without complex medical terminology. The clinician and patient then discuss these options together before coming to a joint decision on which treatment is preferred. So based on that information…

1. How might shared decision-making support patients choose which airway clearance technique may be best for them?
   1. How do you think using shared decision making to support patients’ choice of airway clearance technique might influence their adherence?
2. What do you feel would be the pros and cons of a shared decision-making approach if this was adopted into bronchiectasis clinical practice?
   1. ***Removal of prompt. PAG felt the prompts may influence what participants say. For example if I said “length of appointments?” They might just say “oh yes” . It is a leading prompts and would lead to confimational bias***
3. What do you feel are the key pieces of information to share with patients around their airway clearance techniques?
   1. What type of format do you think information should be shared with for patients? Paper, digital? Both other? Multi-lingual? Pictures? Videos?
   2. If sharing information on a range of airway clearance techniques, how many choices/ACTs do you think would be acceptable?
   3. Prompt if not covered: Would a video showing each/a range of airway clearance techniques/ breathing exercises be useful?
4. Have you had any shared decision-making training to date?
5. Thinking about how you and other bronchiectasis physiotherapists might learn about shared-decision making and how to use it in clinical practice to support patients’ choice of airway clearance, what method(s) of training do you feel would work for this?
   1. F2F workshops? Online tutorial? Webinar? Peer to peer? One “champion” at the trusts and dissemination through in-service/ micro teaching?
   2. What do you feel would be the key things to include in this teaching/training package for shared decision making on airway clearance techniques?
6. Overall, do you think the use of SDM to support patients choose their ACTs would be acceptable? (Y/N).
7. What else do you feel would help support patients to optimise their concordance with airway clearance for their bronchiectasis?
