# Supplementary material for: Qualitative exploration assessing the acceptability of shared decision-making for prescribing airway clearance techniques in adults with bronchiectasis
Source: BMJ Open. 2026 Jul 28;16(7):e119884. doi: 10.1136/bmjopen-2026-119884 (PMC13423159; doi:10.1136/bmjopen-2026-119884)
Supplement: online supplemental file 3 [file bmjopen-16-7-s003.docx]

| **Theme 1: Adherence** | HFG2: “because if the patient felt listened to and they’d gone through all the different options. You’ve asked them what they think would fit better in their lifestyle rather than me making a decision by myself on their behalf of what I think would be best that would fit in their lifestyle…I would say the more you explain, the more you get buy-in, the better adherence you get.”  HI06: “Better patient outcomes, better patient engagement, people are more likely to continue with their airway clearance in my experience if they’ve had some say over what they’re doing, and they’re more likely to come back. You never know, we [physiotherapists] might learn something or a way to do them [ACTs] more”  HI05: “I think, particularly with some of the younger patients, those coming to us from paeds whose adherence is maybe not great- I’m not necessarily talking, now, about new patients, I suppose. They might be new to me, but may have seen a physio before…and actually, in that moment, giving them a choice of whatever like an adjunct or a different one [ACT]…I think they’d be more engaged and stick with it, because they feel like they’re going away with that independent choice. You never know they might even turn up to our service [adult bronchiectasis] again which is rare.” |
| --- | --- |
| **Theme 2: Cultural challenges** | PI08: “It depends. I mean, it depends on who you're talking to using it [SDM]. I mean, you're easy to talk to, historically though, really, you've got somebody who's really dominant and saying, "Tch, tch, tch, tch, that's how you do it,". If they're really officious, I wouldn't like that. It wouldn’t work. You know, that would be, like, sort of, enforced, rather than decided. They would think they were sharing, if that makes any sense. But they aren’t, they are just telling me.”  HI02: “Medicine, generally, is based on a hierarchy, isn’t it? So, actually…if someone has had something since they were very young like bronchiectasis which the majority we could probably agree on that get it when they’re younger, whooping cough, something like that, that they’ve been dictated to in a long time of their life, they wouldn’t talk to the doctor, just listen. For them getting in a mindset of shared care is quite different.” |
| **Theme 3: Education** | HI07 “I would expect it [SDM] to include a lot of what I do anyway like education. So one of the reasons why they [patients] only ever leave with active cycle on a first session is because all of the rest of it [consultation] is education about their condition. That’s key in the first session”  HI05” But I think in terms of involving patients in that discussion with it [SDM] and trying to educate them, there are no real other cons to that, that I can think of. It might take a bit longer than just shoving something [an ACT] at someone, without talking to them about it, but it’s not going to work out in the long run. If they know a few, then switching would be easier for exacerbations or in other appointments”  HI09: “The individual doesn’t have the experience that we have about airway clearance so they, perhaps, are floundering a little bit and think, “You know what, I don’t actually know what the best thing to do is. I don’t really need to know why it works, I’m new to all this, I just want you to tell me how to clear my chest better.”  HFG3: “it’s all well and good saying that we need to be doing these things and we should be doing this, in certain areas in healthcare it works beautifully. For example, the surgery, you either have surgery or this, and these are the consequences of both, its binary, like mortality risk, its black and white, its simple for patients. In therapies is a little different. Especially in respiratory because you know what they need to do, but it’s actually their ability to understand what’s going on, because a lot of them are in denial of respiratory conditions, whether that’s breathing pattern or airway clearance, because it’s something that they just don’t really get. I just don’t think it’s- It’s as easy to say that it should be blanketed across everywhere”. |
| **Theme 4: Empowerment** | HFG1: “I think that the reverse of the overwhelming is the empowering. They’re taking control of, “I'm doing this to treat my bronchiectasis.” So, you’ve given them the information and then they’re saying, “Well, it’s the Acapella that works for me,” or, “The ACBT that works for me.”  PFG1: “yeah, it [SDM] would use the time wisely, being made to feel comfortable enough to ask the questions, that sits well with me…You kind of feel then a little bit more empowered that you're taking a bit of control over your health.” |
| **Theme 5: Engagement** | PFG1: “I'd like to think we've got away from the, “The doctor is the expert, so you don't question him. He will tell you what to do, and you do it, or you don't,” or whatever. But I do think people…I think there's still some of that around. Well doctors and you lot [physiotherapists]. I like to think it's getting less, but I'm not sure. I’m not sure you would do it [use the SDM intervention]”  PFG1: I don’t mind booklets you know, but could this (SDM) be online to use? To compare clearance exercises [ACTs], I mean…I’ve used Be Happy [a UK patient bronchiectasis group] and the European Lung Clinic websites and things like that…I have found it's been most informative about the clearance exercises [ACTs].”  HI05: “I would hope, by having the shared decision, that you’re able to just talk things through with them [patients]. We’re always going to push that they need to do their airway clearance, in one way or another. I think there only has to be positives, really, in involving your patient in their decision and in their treatment.”  PI03: “… helping them decide what their priorities are and how to prioritise that within their day- life management, along with their other priorities. Timescales, time involvement, what they need to be able to do those exercises, you know, is it more appropriate to provide an Aerobika and say, “Okay, let's skip the postural drainage and do it sitting in the loo at work at lunchtime”…could that be in there [SDM PtDA/intervention]?”  HFG2: “Then I think there’s probably a group of people who just want to be told what to do and not have to think about it. I think we probably all try and take the approach of shared decision-making, but then there’s probably a group of people who are just like, “I don’t want the added stress of trying to figure out what is best for me.”  PFG1: “The confidence to speak is not something I struggle with, but I can imagine why it would be difficult for others if they’ve not got somebody approachable to talk to about it [SDM]… And the tone of voice can sometimes, you know, make you feel uncomfortable, “Oh, they need to be somewhere else. I'm not important. They've got something else to do. I better not ask, I’m a burden” That happened to me before from a knowledgeable doctor”. |
| **Theme 6: Flexibility** | HI01: “like I said earlier, if there’s something [an ACT] they could use that was more important to the patient…so, perhaps, the inflexibility of it [SDM PtDA], I might find frustrating…and so I don’t know I need a model. I don’t know. What if that ones not there? That might be very arrogant of me.”  PI02: “To be told what [ACT] is on offer, and explain that, you know, one size doesn’t fit all at all. So, yeah, absolutely, flexible to meet our needs and including the negatives and the positives.”  HFG3: “So I think what your original question is, it’s trying to know what your [bronchiectasis] service is doing currently and seeing where you can fit it in, how the staffing is, to then say, “Okay, this is what’s feasible. This is what we can do.” Because the guidelines are great, but it’s difficult to put into practice. Can we use this [SDM]? Maybe, maybe not”  PI07 “It must be difficult for a physiotherapist to adapt each time and be as equally interested in every single patient that comes through the door and adapt to their needs. I can sympathise because of the pressures on the healthcare system, particularly at the moment. This thing [SDM] could help maybe, have those different conversations you know, in a focused but flexible way”. |
| **Theme 7: Health literacy** | PFG1: “It [SDM PtDA] needs to be clear, nothing too clever because it's understanding what's going on, so you can make the choices, where I don't feel that I've had that… enough knowledge, that if I did have that conversation back then, I'm not sure I'd be able to make that individual decision.”  HFG2: “I think the most important thing is to make it [SDM] as simple as possible” |
| **Theme 8: Overwhelming information** | HFG1: “I think one of the cons is overwhelming. We’ve all mentioned that. That they’ll [patients] end up doing nothing because they’ve too much choice.”  PFG2: “I think that two or three different techniques to be tried is what would be useful…Because to be given too much at once, I would find that overwhelming and I would just say, “Oh, forget it,” and not do any of it.”  HI09: “I think giving the new patient too many options, kind of, doesn’t help them focus on what they need to do. Once they have an airway clearance technique established, then, I think options are good.”  HFG3: “I mean, I’m still learning stuff about airway clearance, in terms of all the subtleties of it. So in terms of then having the nuances and knowing enough about that specific thing to choose, beyond a surface level analysis for them, I think that’s the difficulty…We can’t assume that they’ve [patients] got enough health education to make a fully informed choice. That will need thought through” |
| **Theme 9: Participant understanding** | HFG3: “And, actually, as I said earlier, it doesn’t really matter what they do, actually. If someone’s going for an operation, for example, you’d have statistics of this happening if you have the operation and statistics of this if you don’t have the operation, for example. But in ACT, the actual method, we know guidelines actually say what they do doesn’t really matter, it’s doing something. So I suppose I think shared decision, it’s more about educating them on doing something versus not doing anything, more than the actual method that they use.”  HI02: So, share decision-making in principles is that you’re working with an individual, a client, patient, whatever we’re supposed to call them nowadays. We’re working with someone and we’re building a relationship with them to get them to a point where they feel confident and happy in what they’re doing at that time between us [physiotherapists] and the rest of the bronchiectasis team.”  HFG2: “I can see it [SDM] being useful to lay it out. It’s having a conversation with the patient and just finding out what is their expectation, laying out your expectation and then saying, “Okay, this is what we’re trying or what we could try-” It’s like compromising negotiations”.  HI03: “I think…you know, introduction to health coaching or shared decision making and what influences health decisions and health behaviour? Again, that’s something I never touched on until, well, probably ten years into my career in reality. So, I think, that’s part of… that’s a big thing for me as well” |
| **Theme 10: Resources** | PI02: I think you’re up against it, really, because you’ve got that cost, the resources problem... And of course, just doing Pilates, or postural drainage, or ACBT, you could tell people that there are all these things...They’re going to think, “Well, are the resources there for me to try all these things?” And I’m sure the system doesn’t allow, “Oh, well, you can have a Flutter. Oh, that’s £50. Oh, have an Aerobika at the same time another £50, where does it stop? What are you going to stop buying instead? I can’t buy these all myself can I?”.  HI05: “I guess, maybe, the cons would be that, usually, patients will probably want something tangible, and would go for an adjunct, which is more expensive than doing a breathing exercise… Because patients do like to have something to use, so I think it’s probably more expensive…then you’ve got equipment cost is an issue. Not necessarily for us, but obviously the trusts, the hospitals and a wider view. I don’t know if it [SDM] would work in those places” |
| **Theme 11: Supportive of patient preference** | HI06: I guess one of the cons [of SDM] would be that, you know, a patient could choose something that you think might be less effective. But, if they choose that then you, kind of, have to go with it. So, it’s probably just, you know, that catch-22 of giving people options and having to keep your mouth shut even if you think that they’ve chosen the wrong one…”  HFG3: I think, actually, we’re doing a bit of negligence if we don’t say, “Look, I can see you’ve got retained secretions, really you need to use this OPEP device.” And if they say, “I’d rather just do ACBT.” “Well, it’s not really going to be good enough, you’ll get worse, sorry. (Laughter) It’s not enough.” (Laughter) I’m so bossy. (Laughter)”  HFG4: “Yes, we [physiotherapists] know what is optimal, but sometimes that is not what the patient is able to do. They just can't, mentally, or physically, or emotionally, achieve that sort of optimal. It’s not like a medicine you just swallow or inhale, this [ACT] is effortful. Focus on what they [patients] can do”  HFG1: It would be hard to understand how they [patients] would cope with making, what we would see as, the right decision with more options.  Interviewer: Can you explain it a bit?  HFG1: It might overwhelm them, or they might pick what we think is wrong, but then if they pick something, are they more likely to do it? It’s an interesting one. I just want to help them choose the right one.”  PI01: I think that this [SDM] is vital. It’s incredibly helpful for patients like me to feel as though we’re being listened to… making sure that people understand that really what you’re trying to do is everything is tailored personally to have benefit of them…If people don’t feel it’s personal, they won’t buy in. That’s my opinion anyway”. |
| **Theme 12: Time** | HI03: “it's time. If you've got… I get 40, 45 minutes for a patient appointment. So doing everything in 45 minutes can be challenging. Very challenging. The negatives [of using a SDM intervention] are, at the time, because it takes longer, making sure the patient understands, can be challenging.”  HFG1: “Yes. I think a perceived con might be time…but it’s using probably other skills like motivational interviewing, coaching. You know, you learn the technique and then when you become good at it, you become more efficient…. So, I suppose, it could be a con initially, but that might actually have a pro further down the line, like you know, save an appointment or two”  PFG2: “I think a dialogue is important but, with me, I’m not very quick. I’m not very quick and thinking on my feet and responding. How much time do we get? Also, I think by the time you get home, you probably remember only half if it, if that. I do like it written down. Then I would have more time to make a decision…perhaps we could be given it beforehand, I don’t know.”  HI04: “Because the whole time you’re speaking to them your brain’s tick, tick, tick, tick isn’t it, thinking. You don’t do the 45 minutes and think, “Oh what am I going to do now?” The whole time I’m thinking, “Where am I going with this?”… I might say, “This is where I’m thinking,” and if they’re happy I’d do it but if they’re happy I wouldn’t necessarily say, “Or we could do this or we could do that,” and start with that and then move on, if that makes sense?” |
