# Supplementary material for: Qualitative exploration assessing the acceptability of shared decision-making for prescribing airway clearance techniques in adults with bronchiectasis
Source: BMJ Open. 2026 Jul 28;16(7):e119884. doi: 10.1136/bmjopen-2026-119884 (PMC13423159; doi:10.1136/bmjopen-2026-119884)
Supplement: Supplementary file 4 [file bmjopen-2026-119884.reviewer_comments.pdf]

PEER REVIEW HISTORY

BMJ Open publishes all reviews undertaken for accepted manuscripts. Reviewers are asked to complete a checklist review form and are provided with free text boxes to elaborate on their assessment. These free text comments are reproduced below.

ARTICLE DETAILS

Title (Provisional)

Qualitative exploration assessing the acceptability of shared decision making for prescribing airway clearance techniques in adults with bronchiectasis

Authors

McCallion, Paul; Bradley, Judy; Lally, Joanne; Robinson, Lisa; MacGregor, Fiona; De Soyza, Anthony

---

VERSION 1 - REVIEW

**Reviewer**	**1**
**Name**	**Herrero-Cortina, Beatriz**
**Affiliation**	**Universidad San Jorge de Zaragoza**
**Date**	**03-May-2026**
**COI**	**None**

Upon carefully reading your manuscript, I share with you my major and minor concerns, which I hope will help improve its quality. Congratulations on your work and the topic selected.

Please, see below my major concerns

- Bracketing procedure. In qualitative studies, particularly when the same individual facilitates the focus groups or interviews and subsequently analyzes the data, it is essential to implement a reflexivity process (or bracketing) to address potential researcher bias. Could the authors describe how this reflexivity process was conducted in this project? This should be explicitly detailed in the Methods section, and if not adequately addressed, the Discussion should acknowledge and highlight this as a key methodological limitation.

- PAG. It is not entirely clear whether the PAG members participated in the qualitative study or not or only gave suggestions and collaborated with the study design.

- Interviews / Focal groups: It would be highly interesting if the authors could highlight the specific contributions made by the PAG to interviews /focus group design, as this represents one of the manuscript's key strengths.

- SDM: It is not clear to me whether the authors prepared an initial draft of the SDM to use with the physiotherapists and patients, to help familiarize them with the concept and to analyse its acceptability…if yes…this has not been shared in the article…right?

- ACTs: It is surprising that, to reduce the amount of information and make it more engaging, no mention has been made of presenting the different ACTs in an audiovisual format, for example. What did the authors/physios/patients think about this approach?

- Why were only patients and physiotherapists from the UK selected? Could the results have differed with more heterogeneous groups from various regions?

These are my minor concerns

Abstract: Could the authors consider reduce the introduction section? Some of the information appears to be repeated and might benefit from more concise presentation. At the same time, might it be possible to further expand the results section to provide a more detailed of the findings?

Introduction; page 4, line 20. If the aim is to tailor the choice to the patient and offer all ACT alternatives, we should move away from what is "used in the UK or in other regions" and provide the full range of available options.

Introduction; page 4, line 23. It may be better to refer to the meta-analysis rather than stating that there is no rigorous clinical trials, and to cite reference 1 together with reference 5.

Introduction; page 4, line 25. Could the authors also mention that, beyond etiology, different comorbidities are often an important factor to consider when selecting the ACTs in bronchiectasis?

Introduction; I miss a mention in the introduction of how the ACTs could be selected based on their mechanism of action.

Introduction; page 4, line 56. Since your study focuses exclusively on adults, I do not think reference 13 is the best choice

Introduction, last paragraph. Could the authors clarify here that this is a study conducted exclusively in adults clinically stable, given that the findings may differ in paediatric populations?

Methods, page 6, line 22. I suppose the NTM group was from the UK as well?

Methods; In the sections on patient and public involvement and eligibility criteria, the recruitment location is repeated but inconsistently described, could the authors clarify and standardize this detail? Was PCD involved in the process, or not? Is it because the PAG members were included only as collaborators in the study design and not in the interviews / focal groups?

Methods; page 8, line 9. Is it truly necessary to highlight that PM is a PhD student?

Results; page 8. Could the authors present the results separately for patients and physiotherapists regarding who participated in focus group or interviews?... given that (if I have understood correctly) the physiotherapists only participated in focus groups while patients had the option to choose their participation format?

Results; I suggest including some patient quotes in the main text as well, as they often help readers better understand the results.

Results: I appreciate the figure created by the authors; however, it is challenging to identify which domain relates to the topic in this format. Could the authors consider trying a different presentation style?

**Reviewer**	**2**
**Name**	**Williams, Parris**
**Affiliation**	**Imperial College London, National Heart and Lung Institute**
**Date**	**01-Jun-2026**
**COI**	**None**

This is an interesting qualitative study. I do have some comments that need to be addressed before it is ready for publication

1) The background section of your abstract is far too long; this section should be cut significantly.

2) You need to explain in a bit more detail the TFA and why you used this model. I would suggest this goes into your data analysis methods section.

3) Figure 1 needs more explanation within the text. What is it actually representing ect..

4) I would like a bit more information about the critical thematic analysis process. Did both reviewers independently code all transcripts? How many times did they meet to agree upon the final themes?

5) 12 themes within a qualitative analysis are high; I would expect around 4/5. After reading your results section, it highlights that the authors need to conduct a deeper analysis of their data. It seems their analysis is very descriptive, rather than taking a wider look at the data and the social/cultual/medical context it sits in.

VERSION 1 - AUTHOR RESPONSE

Comments from Reviewer: 1

COMMENT

Bracketing procedure. In qualitative studies, particularly when the same individual facilitates

the focus groups or interviews and subsequently analyzes the data, it is essential to implement a reflexivity process (or bracketing) to address potential researcher bias. Could the authors describe how this reflexivity process was conducted in this project? This should be explicitly detailed in the Methods section, and if not adequately addressed, the Discussion should acknowledge and highlight this as a key methodological limitation.

RESPONSE

Thank for you this valid point to consider. We agree that a reflective account should be included in the manuscript. We have therefore added the following within the limitations section in the manuscript (line 386):

> *"The researcher was respiratory physiotherapist by background with an interest in bronchiectasis. There was an aim to balance subjectivity, that is acknowledgment of the researchers interest on the phenomenon of interest (e.g., ACTs and bronchiectasis) and objectivity (e.g., acknowledging personal assumptions that SDM would be helpful when choosing ACTs). However, this positionality may have monopolised ad-hoc questions, prompts or even the tone of voice during interviews which may have influenced participants responses and affected the interpretation and analysis of data captured"*

COMMENT

PAG. It is not entirely clear whether the PAG members participated in the qualitative study or not or only gave suggestions and collaborated with the study design.

RESPONSE

Thank you for this comment. We have added a sentence (line 103) to clarify PAG members were not participants in the study:

> *"These members contributed to the design of the study development and iterations of topic guides (supplementary material 2). They were not participants in the study."*

COMMENT

Interviews / Focal groups: It would be highly interesting if the authors could highlight the specific contributions made by the PAG to interviews /focus group design, as this represents one of the manuscript's key strengths.

RESPONSE

Thank you for this important comment. PAG members supported the writing of the topic guides to support Plain English wording for ease of comprehension for participants. PAG members also contributed to iterations of topic guides after initial interviews/focus groups. We have updated the manuscript to reflect this (line 101):

> *"Members of the group (n=3) were recruited from Asthma and Lung UK (ALUK) and Non-Tuberculous Mycobacterium (NTM) Patient Networks. These members contributed to the design of the study development and iterations of topic guides (supplementary material 2). They were not participants in the study. The results of this study were shared with the PAG to facilitate interpretation of findings prior to the next stage in the fellowship.*

COMMENT

SDM: It is not clear to me whether the authors prepared an initial draft of the SDM to use with the physiotherapists and patients, to help familiarize them with the concept and to analyse its acceptability…if yes…this has not been shared in the article…right?

RESPONSE

Thank you for mentioning this valid point. This study investigated the anticipated acceptability of a using SDM (pre-SDM intervention development stage). However, during the interviews and focus groups, the interviewer read out and shared (either a paper copy or shared screen over a video call) a description of SDM (included in the topic guide – supplementary information 2). At this point in the interviews and focus groups, participants were giving the opportunity to ask any questions on SDM to allow them to provide their

views and opinions for the subsequent questions. We have added the following within the methods section (line 155):

"The concept of SDM was introduced to physiotherapists and patients during interviews. The interviewer read out and shared (either a paper copy or shared screen over a video call) a description of SDM"

COMMENT

ACTs: It is surprising that, to reduce the amount of information and make it more engaging, no mention has been made of presenting the different ACTs in an audiovisual format, for example.
What did the authors/physios/patients think about this approach?

RESPONSE

We thanks the reviewer for this comment. Yes, this sub-theme appeared within the 'Engagement' theme (theme 5). This was previously not included due to the limited manuscript word count. We have therefore replaced one component of the 'engagement' theme to include this (line 232):

"Participants also highlighted that availability of digital content (website or phone application) may support engagement with SDM and possibly influence their ability to perform their chosen ACT:

PFG1: I don't mind booklets you know, but could this (SDM) be online to use? To compare clearance exercises [ACTs], I mean…I've used BE Happy [a UK patient bronchiectasis group] and the European Lung Clinic websites and things like that…I have found it's been most informative about the clearance exercises [ACTs]."

COMMENT

Why were only patients and physiotherapists from the UK selected? Could the results have differed with more heterogeneous groups from various regions?

RESPONSE

Many thanks for bring up this limitation to the study. Yes, only participants in the UK were used. The overall NIHR fellowship was titled “Feasibility and acceptability of using a Shared Decision Making intervention to support patient choice of Airway Clearance Techniques in bronchiectasis physiotherapy consultations in the NHS” – this unfortunately limited our participant group to NHS service users in the UK. We have therefore acknowledged this in the limitation section (line 375):

> *“Additionally, the study only recruited from the UK, results may have differed and potentially been more widely applicable with a more heterogeneous population.”*

COMMENT

Abstract: Could the authors consider reduce the introduction section? Some of the information appears to be repeated and might benefit from more concise presentation. At the same time, might it be possible to further expand the results section to provide a more detailed of the findings?

RESPONSE

Thank you for this important point. The introduction has been significantly shortened to allow all other additional comments from the reviewers and editor to be addressed.

COMMENT

Introduction; page 4, line 20. If the aim is to tailor the choice to the patient and offer all ACT alternatives, we should move away from what is “used in the UK or in other regions” and provide the full range of available options.

RESPONSE

We agree with this statement. We have removed the phase “used in the UK and in other regions”. We did not provide an exhaustive list of ACTs. We did update the manuscript to reflect this (line 58):

“There are a wide range of ACTs used in bronchiectasis including but not limited to manual techniques, a variety of breathing exercises and hand-held devices (ACT adjuncts)”

COMMENT

Introduction; page 4, line 23. It may be better to refer to the meta-analysis rather than stating that there is no rigorous clinical trials, and to cite reference 1 together with reference 5.

RESPONSE

Thank you for this comment. We have updated the sentence and reference as suggested (line 59):

> **"***Recent guidelines and meta-analysis conclude there is no single ACT demonstrating superiority over another* [1 4]*.*

COMMENT

Introduction; page 4, line 25. Could the authors also mention that, beyond etiology, different comorbidities are often an important factor to consider when selecting the ACTs in bronchiectasis?

RESPONSE

Thank you for this important component which may influence ACT choice. We have updated the manuscript (line 61):

> **"***Differing aetiology, multi-morbidities and fluctuating disease status' of patients means a single ACT is unlikely to be appropriate for all patients and superior across the disease trajectory* [5]*."*

COMMENT

Introduction; I miss a mention in the introduction of how the ACTs could be selected based on their mechanism of action.

RESPONSE

Thank you for this observation. We have updated the introduction to include (line 63):

"ACT prescription is complex and prescription patterns differ within and between countries globally. Prescription can be influenced by mechanisms underpinning different ACTs, availability of ACTs and provider experience"

COMMENT

Introduction; page 4, line 56. Since your study focuses exclusively on adults, I do not think reference 13 is the best choice

RESPONSE

Many thanks for this observation. We agree with the comments and have updated the reference accordingly.

COMMENT

Introduction, last paragraph. Could the authors clarify here that this is a study conducted exclusively in adults clinically stable, given that the findings may differ in paediatric populations?

RESPONSE

Thank you for this comment. The clinical stability of patients was not assessed or known at the time of interviews or focus groups. However, we have updated the manuscript to include the word 'adults' to ensure it is clear to the reader the paediatric population was not used in this study (line 83):

"*We conducted an explorative qualitative study to understand bronchiectasis adult patients' and respiratory physiotherapists' anticipated acceptability of a SDM intervention to support patient choice of ACTs in bronchiectasis"*

COMMENT

Methods, page 6, line 22. I suppose the NTM group was from the UK as well?

RESPONSE

Yes, this is correct the NTM group was from the UK. We have updated the manuscript to reflect this (line 113):

> *"Patients were recruited via respiratory charity and patient organisations including ALUK Respiratory Voices, UK NTM Patient Network and the Primary Ciliary Dyskinesia (PCD) Patient Network."*

COMMENT

Methods; In the sections on patient and public involvement and eligibility criteria, the recruitment location is repeated but inconsistently described, could the authors clarify and standardize this detail?

RESPONSE

Thank you for this comment. To clarify, the Patient Advisory Group (PAG) members were recruited from Asthma and Lung UK (ALUK) and UK Non-Tuberculous Mycobacterium (NTM) Patient Networks. This was at the inception of the larger project.

Patients who participated specifically in this study were recruited from ALUK Respiratory Voices, NTM Patient Network and the Primary Ciliary Dyskinesia (PCD) Patient Network. The PCD network was not used to recruit the PAG group at the beginning of the larger project. Recruitment for each of these separate participants are stated under the respective sub-headings. The manuscript has been updated in the two sections.

Patient and public involvement:

"The fellowship had a patient advisory group (PAG). Members of the group (n=3) were recruited from Asthma and Lung UK (ALUK) and United Kingdom (UK) Non-Tuberculous Mycobacterium (NTM) Patient Networks."

And under eligibility criteria:

"Patients were recruited via respiratory charity and patient organisations including ALUK Respiratory Voices, UK NTM Patient Network and the Primary Ciliary Dyskinesia (PCD) Patient Network."

COMMENT

Was PCD involved in the process, or not? Is it because the PAG members were included only as collaborators in the study design and not in the interviews / focal groups?

RESPONSE

Yes, this is correct. As described in the previous comment, the PCD network was not used to recruit the PAG group at the beginning of the larger project. The PCD network was used to recruit patient participants for interviews and focus groups in this study (line 113).

> *"Patients were recruited via respiratory charity and patient organisations including ALUK Respiratory Voices, UK NTM Patient Network and the Primary Ciliary Dyskinesia (PCD) Patient Network."*

COMMENT

Methods; page 8, line 9. Is it truly necessary to highlight that PM is a PhD student?

RESPONSE

The COREQ checklist, and the BMJ journal editor both described the need to state the background of the researcher(s) who conducted the interviews.

COMMENT

Results; page 8. Could the authors present the results separately for patients and physiotherapists regarding who participated in focus group or interviews?... given that (if I have understood correctly) the physiotherapists only participated in focus groups while patients had the option to choose their participation format?

RESPONSE

Thank you for this comment. Both participant groups were offered a choice of individual interview or focus group format. We have updated this in the manuscript under 'data collection' (line 144):

> *"Both physiotherapists and patients were offered a choice. All video calls for interviews and focus groups were conducted from PM's private home or Research*

Team office at Newcastle University. Face to face focus groups or interviews were conducted in a university conference room."

Additionally, we have updated the results section to clarify how many of each group were involved in focus groups and interviews (line 179):

> *"Forty-two people (21 respiratory physiotherapists and 21 patients living with bronchiectasis) participated in either focus groups (n=6; 11 physiotherapist, 8 patients) or interviews (n=22; 9 physiotherapists, 13 patients)."*

COMMENT

Results; I suggest including some patient quotes in the main text as well, as they often help readers better understand the results.

RESPONSE

Many thanks for this valuable comment. To keep within the word count for the journal, we were unable to provide quotes for every theme. However, we have included quotes from both patients and physiotherapists for selected themes throughout the results section (lines, 210, 226, 241, 262 and 279).

COMMENT

Results: I appreciate the figure created by the authors; however, it is challenging to identify which domain relates to the topic in this format. Could the authors consider trying a different presentation style?

RESPONSE

Thank you for this feedback. We have updated figure 1 into a more aesthetically pleasing design. We hope these changes are satisfactory.

Reviewer: 2

COMMENT

1) The background section of your abstract is far too long; this section should be cut significantly.

RESPONSE

Thank you for this important point. We agree. The introduction has been significantly shortened to allow all other additional comments from the reviewers and editor to be addressed.

COMMENT

2) You need to explain in a bit more detail the TFA and why you used this model. I would suggest this goes into your data analysis methods section.

RESPONSE

Many thanks for this comment. We have updated the methods section to include a description of the TFA (line 148):

> *"Topic guides were designed using the TFA questionnaire template by Sekhon et al. (2022). This framework consists of seven constructs: affective attitude, burden, ethicality, intervention coherence, opportunity costs, perceived effectiveness and self-efficacy. These reflect to what extent people receiving or delivering a healthcare intervention find acceptable based on anticipated (pre-intervention) or experienced (during or post-intervention) cognitive and emotional responses (Sekhon et al., 2017)."*

COMMENT

3) Figure 1 needs more explanation within the text. What is it actually representing ect..

RESPONSE

Thank you for this comment. We have added a sentence to improve the clarity of what figure 1 represents (line 187):

"Twelve themes were identified covering all seven domains of the TFA (figure 1). Each generated theme represents a component of acceptability within the pre-defined domains of the TFA"

Additionally, we have updated figure 1 into a more aesthetically pleasing design. We hope these changes are satisfactory.

COMMENT

4) I would like a bit more information about the critical thematic analysis process. Did both reviewers independently code all transcripts? How many times did they meet to agree upon the final themes?

RESPONSE

Thank you for this comment. We have described critical realist analysis in more detail (Line 168)

> *"We used critical realist thematic analysis to analyse the data [25 26]. This aligned with the overall philosophical position of authors wider thesis, critical realism. This approach used qualitative data to explore experiences and views of participants, creating themes and mapping these to an a priori table of constructs outlined by the TFA [16]. Data were analysed independently by PM and FM (PhD student in forensic radiology). After 5 transcripts were coded independently, codes underwent standardization and consolidation by PM and FM. This process occurred again after all transcripts were coded to ensure consistency. Themes were also verified in discussion with full supervisory team. Themes were mapped to all seven constructs of the TFA. Any discrepancies were mediated through discussion between PM and FM."*

Additionally, this piece of work was part of the authors larger PhD fellowship. The authors philosophical position for this PhD was critical realism. This is one reason why critical realist thematic analysis was used. We have therefore added in the methods section (line 169):

> *"This aligned with the overall philosophical position of authors wider thesis, critical realism."*

COMMENT

5) 12 themes within a qualitative analysis are high; I would expect around 4/5. After reading your results section, it highlights that the authors need to conduct a deeper analysis of their

data. It seems their analysis is very descriptive, rather than taking a wider look at the data and the social/cultural/medical context it sits in.

RESPONSE

Many thanks for this salient point. We agree that 12 themes may appear atypical for a thematic analysis. However, as the theoretical framework of acceptability has seven distinct domains, there was an effort to generate themes which mapped to all seven domains. This is one reason why a higher number of themes were generated.

In addition, the study aim was to explore anticipated acceptability of developing a SDM intervention. Many themes generated were subsequently shared with the co-production team (a separate group who co-produced the SDM intervention) to inform the design process. The higher number of themes generated, allowed a more comprehensive description on elements of acceptability which influenced the design and may contribute to future acceptability of the SDM intervention.

To reflect this, we have provided an in-text table (table 1, line 307), and the following text (line 300):

> *"This is the first study exploring the anticipated acceptability of a SDM intervention to support ACT choice in bronchiectasis. Using a deductive analysis aligned to the TFA domains, allowed generation of themes directly associated with factors of acceptability. Themes generated reflected i) potential medical and cultural influences on SDM acceptability (time, engagement, cultural challenges) and ii) expectations and challenges when developing the future SDM intervention (table 1)"*

Table 1. Generated themes and their influence for SDM intervention development

Themes	***Relevance to SDM intervention development***
Education	*Patients and physiotherapist felt a patient decision aid should include information to support education on bronchiectasis and a range of ACTs*

Flexibility	*Physiotherapists concerned patient decision aid may restrict choice of alternative ACTs. This may influence the number of ACTs to include in the patient decision aid*
Health literacy	*Patient and physiotherapists stated they wanted minimal medical jargon in the SDM intervention to support easier readability*
Overwhelming information	*Physiotherapists concerned the patient decision aid may be provide too much information and overwhelm patients. This may influence the volume of desirable but non-essential content in the patient decision aid*
Resources	*Physiotherapists concerned patient decision aid will include multiple ACT adjuncts which they may not be able to provide to patients*

Additionally, as the reviewer suggested, we have updated our discussion to link generated themes to the wider social, cultural and medical contexts (line 331-349).

"The perceived culture of patients using, and professionals working in the NHS appeared to be an influencing factor on the acceptability of SDM. This is separate to the lack of readiness or desire by patients described earlier. There are recognised cultural barriers to SDM in healthcare including perspectives on decision role, personal belief systems, trust in clinicians and historical engagement in medical led decision making. For example, evidence suggests patients from non-western cultural backgrounds appraise their personal decision-making process less positively than Western, Caucasian born patients who are the current demographic majority within the NHS. The assumption with SDM is that every patient has an autonomous choice. This is something that may feel uncomfortable or possibly unwanted in non-western cultures during healthcare consultations.

Studies suggest cultural awareness and inclusion of a wider cultural demographic of stakeholders when developing SDM interventions may strengthen its cultural congruency. Alden, et al. suggest the inclusion of targeted visuals (e.g., fonts, colour or images), sociocultural values (e.g., the importance of involving family members) and linguistics (e.g.,

the ability to translate material into different languages) may increase the perceived relevance of the treatment decision to be made in amongst different cultures. This may also improve the effectiveness of the PtDA compared to one that has 'generic' non-personalised content. However, Bravo, et al. states a balance must be obtained adapting SDM interventions for diverse cultural contexts, without compromising the core essence of SDM."

Thank you for the opportunity to review our manuscript. We hope you find the responses satisfactory, and we feel the changes have greatly improved this manuscript.

VERSION 2 - REVIEW

**Reviewer**	**1**
**Name**	**Herrero-Cortina, Beatriz**
**Affiliation**	**Universidad San Jorge de Zaragoza**
**Date**	**16-Jul-2026**
**COI**	

Thank you for the effort in addressing all the comments and suggestions. I was not able to see the changes made to Figure 1, but I am otherwise in agreement with everything.

Congratulations on the work, and please keep advancing research in this field.
